# Antiviral Activity and Underlying Action Mechanism of Euglena Extract against Influenza Virus

**DOI:** 10.3390/nu13113911

**Published:** 2021-10-30

**Authors:** Ayaka Nakashima, Yuka Horio, Kengo Suzuki, Yuji Isegawa

**Affiliations:** 1 Euglena Co., Ltd., Tokyo 108-0014, Japan; nakashima@euglena.jp (A.N.); suzuki@euglena.jp (K.S.); 2 Department of Food Sciences and Nutrition, School of Human Environmental Sciences, Mukogawa Women’s University, Nishinomiya 663-8558, Japan; 2031761@mwu.jp

**Keywords:** *Euglena gracilis*, Euglena extract, influenza virus, zinc, antiviral effect

## Abstract

It is difficult to match annual vaccines against the exact influenza strain that is spreading in any given flu season. Owing to the emergence of drug-resistant viral strains, new approaches for treating influenza are needed. *Euglena gracilis* (hereinafter Euglena), microalga, used as functional foods and supplements, have been shown to alleviate symptoms of influenza virus infection in mice. However, the mechanism underlying the inhibitory action of microalgae against the influenza virus is unknown. Here, we aimed to study the antiviral activity of Euglena extract against the influenza virus and the underlying action mechanism using Madin–Darby canine kidney (MDCK) cells. Euglena extract strongly inhibited infection by all influenza virus strains examined, including those resistant to the anti-influenza drugs oseltamivir and amantadine. A time-of-addition assay revealed that Euglena extract did not affect the cycle of virus replication, and cell pretreatment or prolonged treatment of infected cells reduced the virus titer. Thus, Euglena extract may activate the host cell defense mechanisms, rather than directly acting on the influenza virus. Moreover, various minerals, mainly zinc, in Euglena extract were found to be involved in the antiviral activity of the extract. In conclusion, Euglena extract could be a potent agent for preventing and treating influenza.

## 1. Introduction

Influenza is an airway infection caused by influenza viruses of three known types—A, B, and C. The most prevalent types (i.e., A and B) comprise two surface glycoproteins, hemagglutinin (HA) and neuraminidase (NA), which are antigens targeted by the host for protective immunity. Influenza virus Type A has 18 subtypes of HA and 11 subtypes of NA, and their varying combinations cause differences in antigenicity. Viruses with different combinations of these glycoproteins are not only widely distributed in humans, but also other hosts such as pigs and birds. Even within the same subtype, the antigenicity of HA and NA can change gradually owing to the accumulation of mutations in the viral genes. Because antigenic mutations (antigenic drift) occur frequently in influenza viruses, they give rise to epidemics almost every year. Generally, after an incubation period of approximately 1–3 days following an infection with the influenza virus, fever (usually a high fever of >38 °C), headache, general malaise, and muscle and joint pains will suddenly ensue. These are typically followed by inflammatory symptoms of the upper respiratory tract, such as cough and nasal discharge, which are alleviated after approximately 1 week. In particular, elderly individuals and patients (regardless of age) with chronic diseases of the respiratory, circulatory, and renal systems; metabolic diseases such as diabetes; and reduced immune function, are known to be more prone to secondary bacterial infections in the respiratory system and to the aggravation of the original disease, which increases the risk of hospitalization and death. In children, influenza can also trigger a combination of otitis media, febrile convulsions, and bronchial asthma.

Currently, vaccines are used to prevent influenza virus infections, whereas antiviral drugs are used to treat the disease. However, there are concerns over the adverse effects of adjuvants in existing influenza vaccines, their inability to respond quickly to new viruses, and the fact that they are less preventive than vaccines against other viruses. Additionally, the adverse effects of antiviral drugs are equally problematic, and so is the emergence of drug-resistant strains [1,2,3,4]. Thus, there is a need to develop new preventive and treatment methods to overcome these problems. In this context, we have focused on the functionality of food products and investigated the mechanism of action of components with antiviral activity.

*Euglena gracilis* (hereinafter Euglena) is a unicellular microalga with both plant and animal characteristics. Because the organism is rich in a variety of nutrients, including vitamins, minerals, amino acids, and fatty acids, it is used as both a dietary supplement and a general health supplement [5]. Thus far, it has been confirmed to be effective in providing relief against immune-related symptoms, such as atopic dermatitis [6] and rheumatoid arthritis [7]. In a previous study, mice that were fed food containing Euglena powder for 2 weeks and subsequently infected with the A/Puerto Rico/8/34 (H1N1) strain of the influenza virus showed improved survival rates and reduced virus counts in the lungs [8]. Based on the observed cytokine production pattern in the mice, it was surmised that the microalgae may have contributed to the elimination of the virus by mobilizing the mechanisms of systemic immunity. However, the direct mechanism through which Euglena inhibits the virus in cells has not been elucidated. In this study, we aimed to investigate the anti–influenza viral activity and the mechanism responsible for the water-soluble components of Euglena used in the previous study [8] by in vitro experiments.

## 2. Materials and Methods

### 2.1. Cell Lines and Viruses

Madin–Darby canine kidney (MDCK) cells were grown in Eagle’s minimum essential medium (MEM; FUJIFILM Wako Pure Chemical Industries, Ltd., Osaka, Japan), containing 7% fetal bovine serum (FBS). H1N1 [A/Puerto Rico/8/34 (A/PR/8/34), A/New Caledonia/20/99, A/Beijing/262/95, A/Suita/6/2007, A/Suita/114/2011, A/Osaka/2024/2009, A/Osaka/71/2011], and H3N2 strains (A/Sydney/5/97, A/Suita/120/2011) of influenza virus Type A were used in the experiments along with Type B strains (B/Nagasaki/1/87, B/Shanghai/261/2002). Each virus culture was diluted in serum-free MEM, containing 0.04% bovine serum albumin (BSA, fraction V; Sigma-Aldrich, St. Louis, MO, USA), and then used to infect cells at a multiplicity of infection (MOI) of 0.001 for 1 h at 37 °C. The medium was then removed and replaced with the serum-free Dulbecco’s modified Eagle’s medium (DMEM; FUJIFILM Wako), containing 0.4% BSA and 2 µg/mL acetyl trypsin (Merck Sigma-Aldrich, St. Louis, MO, USA), for the remaining infection period.

### 2.2. Preparation of Euglena Extract

Euglena powder was obtained from euglena, Co., Ltd. (Tokyo, Japan). To prepare the extract [9], 0.5 g of the Euglena powder was suspended in 20 mL of water and the suspension was heated at 95 °C for 2 h. The insoluble fractions were then removed by centrifugation, and the supernatant was collected and passed through a 0.45-µm filter purchased from Merck Millipore Co., Ltd. (Billerica, MA, USA). The sterile supernatant, designated as 100% Euglena extract, was diluted to the required concentration with the DMEM before being added to the cells. The Euglena extract was stored at 4 °C until further use.

### 2.3. Search for Components Associated with the Effect of Euglena Extract

Liquid chromatography-mass spectrometry (LC/MS) measurement and analysis were performed according to a previously described methods [10,11]. The carbohydrate concentration was measured following the method reported by Suzuki et al. [12]. Euglena extract was treated with exo-1,3-β-D-glucanase (Neogen Chemicals Ltd., MI, USA.) or β-1,3-glucanase (Thermo Stable Enzyme Laboratory Co., Ltd., Kobe, Japan). Polyvinylpolypyrrolidone (PVPP; Sigma-Aldrich, St. Louis, MO, USA) treatment was used to remove polyphenols from Euglena extract. The chelating agents ethylenediaminetetraacetic acid (EDTA; FUJIFILM Wako, Osaka, Japan) and ethylene glycol tetraacetic acid (EGTA; FUJIFILM Wako, Osaka, Japan) were added to trap metal ions. A cation exchange resin (CER; GL sciences Inc., Tokyo, Japan) was used to remove metal ions from Euglena extract. The samples were treated twice to remove approximately 99.4% zinc. The zinc concentration was determined using a zinc assay kit (Metallogenics, Tokyo, Japan), according to the manufacturer’s instructions.

### 2.4. Viral Titer Determination in the Presence of Euglena Extract

The effect of Euglena extract on the viral titer was determined following a previously described procedure, with some modifications [13]. Briefly, 1 × 10^5^ MDCK cells in 500 µL of MEM containing 7% FBS were cultured in each well of a 24-well plate (Thermo Fisher Scientific, Waltham, MA, USA) for 24 h at 37 °C. The confluent monolayer of cells was then rinsed twice with serum-free MEM. The virus culture was diluted to an MOI of 0.001, and then incubated with the cells for 1 h at 37 °C. Thereafter, the infected cells were rinsed once with serum-free MEM and then cultured in DMEM containing Euglena extract (500 µL/well). After 24 h, the supernatants were collected as influenza virus samples and used in the focus-forming reduction assay (FFRA).

### 2.5. Focus-Forming Reduction Assay to Determine Viral Activity

A focus-forming reduction assay (FFRA) was performed following a previously described method [13,14], with slight modifications. Briefly, MDCK cells (approximately 10^4^ cells/well) were seeded in 96-well flat-bottom plates (Corning Inc., New York, NY, USA) and incubated at 37 °C under 5% CO_2_ to form monolayers. On the following day, the viral solution was serially diluted 10-fold in 96-well round-bottom plates (Thermo Fisher Scientific, Waltham, MA, USA) with MEM containing 0.04% BSA. The MDCK cells in the 96-well flat-bottom plates were washed twice with serum-free MEM, and then, 30 µL of each viral dilution was added to each well and the plates were incubated for 1 h at 37 °C. After removing the viral solutions, the cells were washed with serum-free MEM, covered with 100 µL of an MEM mixture (equal volumes of FBS-free MEM and Avicel^®^ RC-591 NF; FMC Health & Nutrition) containing 0.4% BSA, and incubated for 18 h at 37 °C. The supernatant was removed, and the cells were first washed with serum-free MEM and then fixed with absolute ethanol at ambient temperature for 10 min. Ethanol was then completely removed. The cells that were not stained and immediately stored at −80 °C.

Immunostaining was carried out by adding 50 µL of murine monoclonal anti-HA antibody (C179 for Type A H1N1 viruses [15], F49 for Type A H3N2 viruses [16], and 7B11 for Type B viruses [17]) and a horseradish peroxidase-conjugated goat anti-mouse IgG antibody (Merck KGaA, Darmoshtat, Germany) to the cells. The peroxidase reaction was then allowed to develop for 30 min according to the procedure described by Graham and Karnovsky [18], using 0.1% H_2_O_2_ and 0.3 mg/mL 3,3′-diaminobenzidine tetrahydrochloride (FUJIFILM Wako) in phosphate-buffered saline. After this reaction, the cells were rinsed with water and dried using a hair dryer. The number of foci in the immunostained infected cells was determined using an inverted light microscope (EVOS Life Technologies, Thermo Fisher Scientific, Waltham, MA, USA).

### 2.6. Time-of-Addition Assay: Pretreatment and Posttreatment Schedules

A time-of-addition assay was performed following a previously described procedure, with some modifications [13]. Briefly, 1 × 10^5^ MDCK cells in 500 µL of MEM containing 7% FBS were plated in each well of a 24-well plate and incubated for 24 h at 37 °C. The monolayers were then rinsed twice with serum-free MEM and infected with A/PR/8/34 (MOI = 0.01) for 1 h at 37 °C. Thereafter, the cells were rinsed twice with serum-free MEM and incubated in DMEM for 24 h at 37 °C. DMEM containing 1 mg/mL Euglena extract, which was approximately ten times the median inhibitory concentration (IC_50_) (Table 1), was added at the following time points: within the 24 h period before infection (−24, −20, −16, −12 to −1 h, pretreatment); between 1 h before infection and the time of infection (−1 to 0 h, adsorption); and between 0 and 4 h, 4 and 8 h, or 0 and 8 h after infection (replication). The cell monolayers were rinsed twice with serum-free MEM after each incubation period, and the medium was replaced with fresh medium. The cells were also incubated with Euglena extract for 12, 16, 20, and 24 h after viral infection (0 to 12, 16, 20, 24 h, posttreatment). The infected cells were then frozen at −80 °C and subjected to two freeze–thaw cycles before determining the viral titer using the FFRA.

### 2.7. Cell Viability Determination

Cell viability was determined using the MTT-based colorimetric assay Cell Proliferation Kit I (F. Hoffmann–La Roche Ltd., Basel, Switzerland). The cytopathic effects in the virus-infected cells treated with various concentrations of Euglena extract were observed under a microscope (EVOS Life Technologies, Thermo Fisher Scientific, Waltham, MA, USA).

### 2.8. Statistical Analysis

The data were analyzed using an unpaired t-test and analysis of variance with the Tamhane T2 test with SPSS version 24.0 (SPSS, Inc., Chicago, IL, USA). The virus titers determined in the time-of-addition and antiviral assays were analyzed using the Student’s t-test in Excel-Toukei version 6.0. Values are presented as mean ± standard deviation. Statistical significance was set at *p* < 0.05.

## 3. Results

### 3.1. Growth Inhibition of Influenza Viruses by Euglena Extract

**Figure 1:**
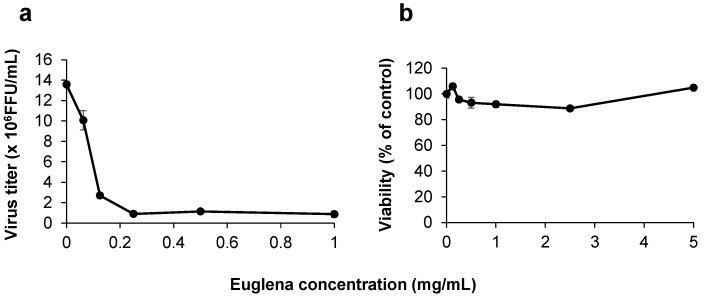
Anti-influenza effect of Euglena extract: (**a**) Viral titer in Madin–Darby canine kidney (MDCK) cells infected with influenza virus strain A/PR/8/34, as determined using the focus-forming reduction assay at 24 h after infection; (**b**) viability of MDCK cells infected with strain A/PR/8/34 after adding Euglena extract. The data are from three independent experiments and the error bars indicate standard deviation (*n* = 3). FFU: focus forming units.

The effect of Euglena extract on A/PR/8/34 (H1N1) growth was verified using the FFRA. This extract inhibited the infection of the influenza virus in MDCK cells in a concentration-dependent manner with an IC_50_ (the concentration at which the extract inhibits the virus by 50%) value of 0.11 mg/mL (Figure 1a). Additionally, no cytotoxicity was observed up to 5 mg/mL extract (Figure 1b).

Next, the antiviral effect of Euglena extract on various strains of Type A and B influenza virus was investigated. As shown in Table 1, the extract suppressed the infection of all tested strains, with IC_50_ values in the range of 0.05–0.11, 0.04–0.08, and 0.05–0.06 mg/mL for the H1N1, H3N2, and Type B strains, respectively. There were no significant differences in the IC_50_ values among the H1N1 strains (*p* > 0.05). Euglena extract also inhibited the growth of oseltamivir-resistant (A/Osaka/2024/2009 and A/Osaka/71/2011) and -susceptible (A/PR/8/34, A/New Caledonia/20/99, A/Suita/6/2007, and A/Suita/114/2011) strains, with similar IC_50_ values. Amantadine-resistant Type B strains also suppressed infection [19,20] (Table 1). These results suggest that the extract might exhibit an antiviral activity even against emerging oseltamivir and amantadine-resistant strains.

### 3.2. Stages of Influenza Growth Inhibited by Euglena Extract

**Figure 2:**
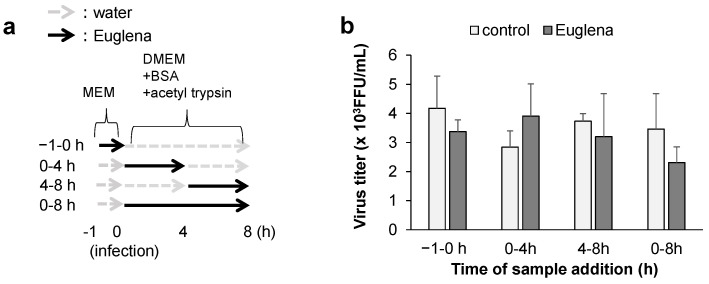
Effect of Euglena extract on the virus titers during its growth cycle. (**a**) Protocol. (**b**) Viral titer in Madin–Darby canine kidney (MDCK) cells infected with influenza virus strain A/PR/8/34, as determined using the focus-forming reduction assay at 8 h after infection. The duration of exposure following the addition of Euglena extract to Madin–Darby canine kidney (MDCK) cells infected with influenza virus strain A/PR/8/34 reflects the growth stage as described in the Methods section. The error bars indicate standard deviation (*n* = 3); no significant difference was observed between the extract-treated cells and control cells. FFU: focus forming units.

Next, we conducted a time-of-addition assay to examine the stage of the influenza virus growth cycle that was inhibited by Euglena extract. One cycle of viral (A/PR/8/34) growth in the cells lasted for 8 h. Figure 2a shows the time points of extract addition, where 0–4 h corresponds to the adsorption phase and the first stage of culture, 4–8 h corresponds to the latter stage, and 0–8 h corresponds to the entire culture period. The antiviral activity of Euglena extract based on this schedule is shown in Figure 2b. The results of the time-of-addition assay showed no significant differences within 8 h.

### 3.3. Strengthening of the Defense Mechanism of Infected Cells by Euglena Extract

**Figure 3:**
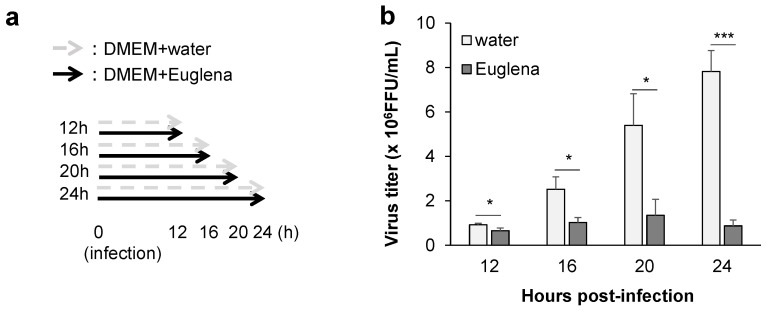
Dependence of the anti-influenza effect of Euglena extract on the treatment duration. (**a**) Protocol. (**b**) Virus titer following the incubation of Madin–Darby canine kidney cells with Euglena extract for 12, 16, 20, and 24 h after viral infection. The error bars indicate standard deviations (*n* = 3); * *p* < 0.05, *** *p* < 0.001. FFU: focus-forming units.

Despite the inhibition of viral growth, the results of the time-of-addition assay (Figure 2a) indicated that the inhibitory effect of Euglena extract was not significantly high (at 60%) throughout the virus culture period (0–8 h after virus infection) (Figure 2b). Therefore, we hypothesized that the addition of the extract for a long period would likely increase its inhibitory effect. Cell supernatants were collected at 12, 16, 20, and 24 h after treatment to measure the virus titer (Figure 3a). We found that viral growth was inhibited significantly at 12 h posttreatment (*p* < 0.05) and confirmed that the inhibitory effect improved even more significantly from 16 h onward (Figure 3b).

**Figure 4:**
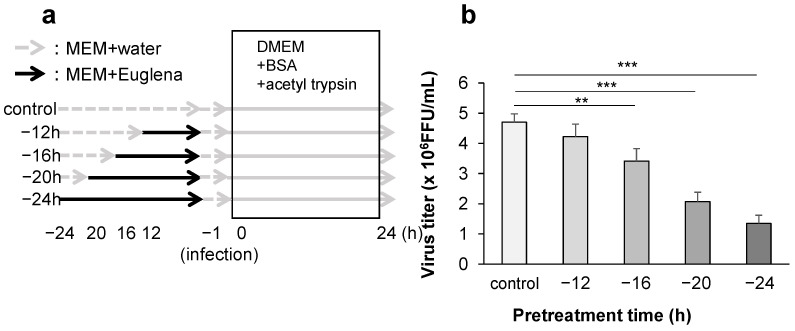
Inhibitory effect of pretreatment with Euglena extract on influenza virus growth. (**a**) Protocol. (**b**) Virus titer following the incubation of Madin–Darby canine kidney cells with Euglena extract for 12, 16, 20, and 24 h before viral infection. The cells were infected with the virus after the extract-containing medium had been replaced. The error bars indicate standard deviations (*n* = 3); ** *p* < 0.01, *** *p* < 0.001. FFU: focus-forming units.

Additionally, the effect of Euglena extract addition to the cell culture before infection with the influenza virus was examined. At 12, 16, 20, and 24 h pre-infection, the medium was replaced with Euglena extract-containing medium, and the cells were cultured. The cells were then washed with serum-free MEM and infected with the virus. After 24 h, the viral titer in the media was measured using the FFRA (Figure 4a). Compared with that in the control cells with no Euglena extract, the virus titer in the cells cultured in the presence of the extract for 16 h before viral infection decreased significantly. This confirmed that the inhibitory effect of the extract improved more significantly when added 20 and 24 h before viral infection (Figure 4b). These results suggest that the viral growth-suppressing effect of Euglena extract may be achieved through an enhancement of the defense mechanism of the infected cells rather than through its direct action on the influenza virus.

### 3.4. Determination of Euglena Extract Components Involved in Growth Inhibition of the Influenza Virus

**Figure 5:**
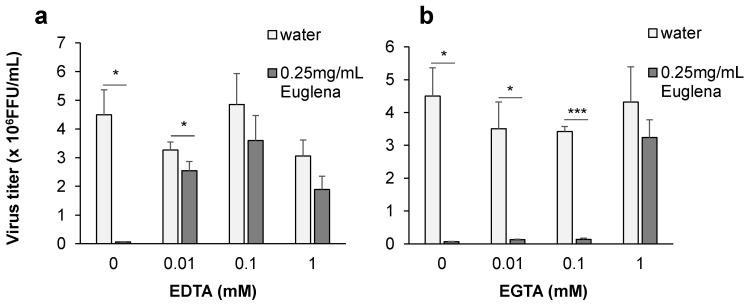
Determination of Euglena extract components involved in influenza virus growth inhibition. Madin–Darby canine kidney (MDCK) cells were cultured 24 h before virus infection with (**a**) EDTA-treated Euglena extract, (**b**) EGTA-treated Euglena extract. Cells were infected with virus after exchanging medium, and then the virus titer was determined. Error bars indicate standard deviations (*n* = 3). * *p* < 0.05, *** *p* < 0.001. FFU: focus-forming units.

We conducted an experiment to determine the components involved in the antiviral activity of Euglena extract. Using the phenol sulfate method, we determined the carbohydrate concentration in Euglena extract to be 0.53%, whereas that in the original Euglena powder was approximately 30–40%. As the anti-influenza virus effect of paramylon as β-glucan has been shown in vivo [8], we treated Euglena extract with two kinds of β-glucanases to determine whether the activity of β-glucan in Euglena extract disappeared in vitro. However, β-glucanases treatment did not alter the antiviral effect of the extract. We then removed polyphenols from the extract via PVPP treatment; however, this treatment did not attenuate the antiviral effect of Euglena extract either. Based on these results, the examined secondary metabolites of Euglena extract did not appear to be active ingredients. Next, we treated the extract with EDTA and EGTA, which are chelating agents for metal ions. Consequently, the antiviral effect of Euglena extract was attenuated, suggesting that metal ions contained in Euglena extract are among the components involved in its inhibitory effect (Figure 5a,b). EGTA, which is known to inhibit low molecular weight minerals such as Mg inhibited the anti-influenza virus activity of Euglena extract at high concentrations. On the contrary, EDTA, which is known to inhibit minerals with molecules larger than Ca, inhibited the anti-influenza virus activity of Euglena extract, even at low concentrations. EGTA has a strong affinity for Mg, Ca, and Cd, but its reactivity with other metals is very weak and limited, whereas EDTA forms complexes with monovalent to tetravalent metals such as Ca, Fe, Cu, Zn, and Ag and is highly reactive with many metals. EDTA was more reactive than EGTA, suggesting that metals other than Mg, Ca, and Cd are involved in the reaction. Zinc is necessary for the growth of Euglena, is known to be stored in its cells, and suppresses the growth of viruses [21,22,23].

**Figure 6:**
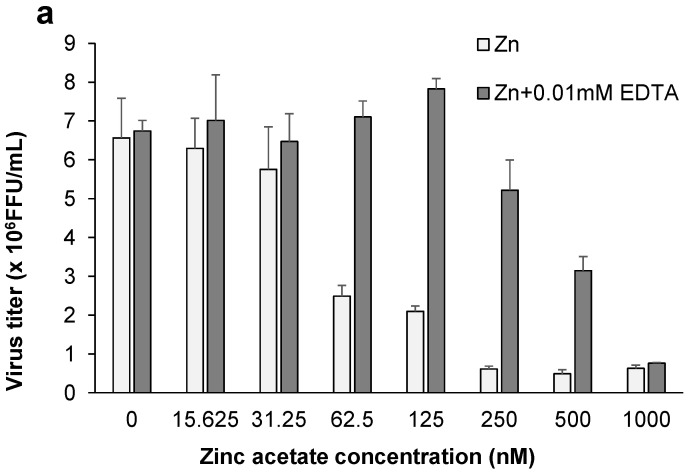

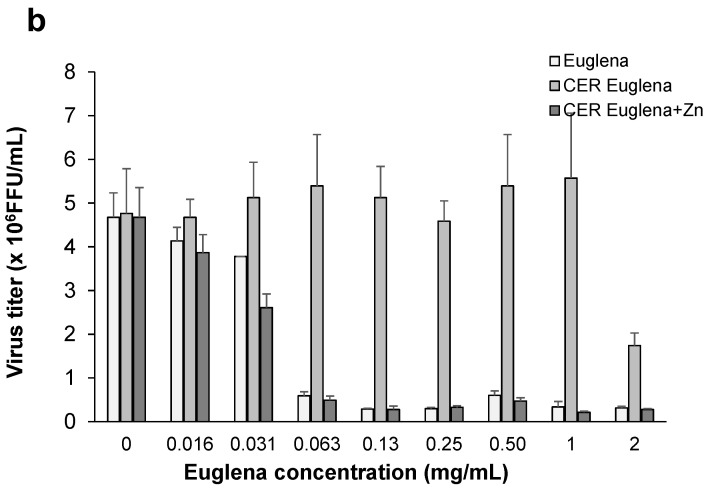
Involvement of zinc in Euglena extract. Madin–Darby canine kidney (MDCK) cells were cultured 24 h before virus infection with (**a**) zinc acetate, (**b**) Euglena extract, CER-treated Euglena extract, and CER-treated Euglena extract + zinc acetate. Cells were infected with virus after exchanging medium, and then the virus titer was determined. The respective zinc concentrations are shown in Table 2. Error bars indicate standard deviations (*n* = 3). CER: cation exchange resin, FFU: focus-forming units.

In this study, we tested the anti-influenza activity of zinc acetate, and confirmed its inhibitory effect on the growth of the influenza virus (Figure 6a). The IC_50_ of zinc acetate was 57.65 ± 10.27 nM. Using CER, we compared the antiviral activity of Euglena extract from which metal ions were removed, with and without zinc acetate. The sample with zinc acetate showed the same antiviral activity as Euglena extract itself (Figure 6b). The respective zinc concentrations are shown in Table 2. Thus, zinc is considered one of the metal ion components in Euglena extract that is essential for the inhibitory effect of the extract.

## 4. Discussion

In this study, we evaluated the anti-influenza effect of Euglena extract using MDCK cells infected with various strains of the influenza virus and confirmed that the extract inhibited the infection of all tested strains in the cells. In particular, we found that Euglena extract can be effective against oseltamivir-resistant virus strains, such as A/Osaka/2024/2009 and A/Osaka/71/2011, suggesting that it inhibits the growth of influenza viruses by a mechanism different to that of oseltamivir. However, there were no significant differences in the IC_50_ values among the various virus strains studied. Thus, the inhibitory activity of Euglena extract on viral replication is predicted to show no viral specificity; this is different from amantadine, which is effective against the influenza Type A virus strain but not against the Type B strain [24].

When the virus attaches to the cell membrane, it is taken up by endocytosis and the RNA is released from the viral particles into the cytoplasm, from where it migrates to the nucleus for replication and transcription; thereafter, the viral proteins and viral genomes are synthesized [25,26]. When the viral components are aligned, virion particles agglomerate near the cell membrane and are released from the cell through the activity of neuraminidase, which is the budding phase of the virus growth cycle. One growth cycle lasts for 8 h, and it is possible to examine the viral process that is inhibited by any substance.

Relenza and Tamiflu, which have become mainstream anti-influenza drugs, inhibit neuraminidase and, hence, the viral budding phase. In contrast, Xofluza, which was launched recently, inhibits viral RNA replication. Here, Euglena extract did not affect the virus replication cycle, and cell pretreatment or prolonged treatment of infected cells reduced the virus titer. The antiviral effect of Euglena extract remained potent even after 16 h of extract pretreatment, which was followed by infection. This result suggests that the extract interacts with the defense mechanisms of the host cell and does not act directly on the influenza virus.

Euglena is a microalga that contains a variety of nutrients, such as vitamins, minerals, amino acids, and fatty acids. It also accumulates insoluble β-glucan (viz., paramylon) as a storage polysaccharide. In mouse infection experiments, oral intake of Euglena has been shown to alleviate the symptoms of influenza virus infection, mainly through the contribution of paramylon [8]. In other studies, the intake of paramylon by mice increased the production of interferon-beta in the blood on Day 3 of viral infection. It has been proposed that insoluble paramylon is recognized by dectin-1 (the primary β-glucan receptor expressed in intestinal immune cells, such as dendritic cells and macrophages), resulting in its intracellular uptake and subsequent promotion of secretion of cytokines through the activation of tyrosine-protein kinase SYK and transcription factor nuclear factor-kappa B [27,28].

The extract produced from the dissolution of Euglena powder in hot water is composed of water-soluble components, from which insoluble paramylon is considered excluded. Using the phenol sulfate method, the carbohydrate concentration in Euglena extract was determined to be 0.53%, whereas that in the original Euglena powder was approximately 30–40%. However, to rule out the possibility that a small amount of glucan was involved, we treated Euglena extract with beta-glucanase to determine whether it would affect the antiviral activity and found that it did not. This finding suggests that beta-glucan is not involved in the antiviral activity of Euglena extract.

Recently, it has been confirmed that Euglena extract suppresses lung cancer symptoms in mice by stimulating host immunity [29]. Thus, it is possible that components other than paramylon in Euglena may be involved in such immune mechanisms. The results of our study corroborate the possibility that multiple components are involved in the multiple mechanisms underlying the antiviral effects of Euglena.

In general, polyphenols exhibit antiviral activity [30]. However, our experiments revealed that polyphenols contained in Euglena extract were not involved in the antiviral effect of the extract. As there was no activity with β-glucans and polyphenols, we examined minerals. Zinc is necessary for the growth of Euglena and is known to be stored in cells. It accelerates the induction of Type 1 interferon receptors compared with other metals [31]. Furthermore, zinc shows antiviral activity [21,22,23]. In our study, the addition of zinc acetate inhibited the growth of influenza viruses in a concentration-dependent manner. Moreover, the addition of zinc acetate to de-metalized Euglena extract restored the influenza virus inhibitory activity of the extract. In 0.031 mg/mL Euglena extract, the zinc concentration was 111.6 nM (Table 2). At 125 nM, when only zinc acetate was added (Figure 6a), the anti-influenza virus activity was similar to that of 0.031 mg/mL Euglena extract (Table 2). It can be inferred that zinc is responsible for this activity. On the contrary, when 2 mg/mL membrane-treated Euglena extract was added, the zinc concentration was 44.06 nM. Figure 6a shows that the addition of zinc acetate alone (31 nM) did not inhibit influenza virus growth as much as membrane-treated Euglena extract, which has an equivalent zinc concentration. In other words, the membrane-treated Euglena extract, which seems to have an equivalent zinc concentration, has a higher influenza virus inhibitory activity than zinc alone.

Ionophores, for example, are required for the cellular uptake of zinc [21], and it is possible that Euglena contains substances that act similar to ionophores, which may be present in Euglena extract at higher concentrations. Ionophores bind zinc to some extent, which may have resulted in the anti-influenza virus activity of the membrane-treated extract at high concentrations. In other words, the effect of zinc acetate was retained in normal Euglena extract because of the presence of free zinc, but in CER Euglena, the effect of zinc bound by ionophores could be demonstrated by removing free zinc by membrane treatment. This suggests that substances other than zinc are also involved in the anti-influenza virus activity of Euglena. The MDCK cells used in this experiment are said to take up zinc relatively easily, but it has been suggested that primate cells such as Vero-E6 cells require ionophores for zinc uptake [21]. Thus, the further consideration of ionophores may be important. A limitation of this study is that although the used MDCK cells are commonly employed in virus research, cell types of other origins should also be investigated. In addition, further studies are needed to confirm the pharmacokinetics of zinc in orally administered Euglena powder and/or Euglena extract in order to investigate the efficacy of zinc in Euglena in vivo.

## 5. Conclusions

Our study showed that Euglena extract reduced the virus titer in vitro, with a more potent inhibitory activity when the host cells were pre-exposed to the extract, indicating that Euglena components may interact with the defense mechanisms of the host cell. Our results show that Euglena and paramylon does not only eliminate viruses through systemic immunity, but that some components in Euglena (particularly zinc) may also have a direct effect on cellular defense mechanisms. Furthermore, our findings indicate that the extract inhibits the growth of influenza viruses by a mechanism not observed in existing drugs and that the inhibitory effect was nonspecific for Type A and B strains. Thus, the extract could be a potent therapeutic option for infections caused by newly mutated influenza virus strains.

## Figures and Tables

**Table 1:** Effect of Euglena extract on the infection of various types of influenza viruses.

Type	Name of Virus	IC_50_	
		Euglena Extract (mg/mL)	Oseltamivir (ng/mL) [13]
A/H1N1	PR/8/34 **	0.11 ± 0.02	0.45 ± 0.01
	Osaka/2024/2009 *	0.10 ± 0.01	180 ± 59
	Osaka/71/2011 *	0.09 ± 0.02	259 ± 75
	Suita/6/2007	0.07 ± 0.01	ND
	Suita/114/2011	0.08 ± 0.01	ND
	New Caledonia/20/99 **	0.05 ± 0.03	0.38 ± 0.18
A/H3N2	Sydney/5/97	0.08 ± 0.01	ND
	Aichi/2/68 **	0.04 ± 0.01	0.93 ± 0.78
B	Nagasaki/1/87 **	0.05 ± 0.00	6.10 ± 1.19
	Shanghai/261/2002	0.06 ± 0.01	ND

* Oseltamivir-resistant, ** oseltamivir susceptible, ND: not determined.

**Table 2:** Zinc concentration in each Euglena extract. CER, cation exchange resin.

Euglena Concentration (mg/mL)	0	0.031	0.063	0.13	0.25	0.5	1	2
Zn (nM) in Euglena	0	111.6	223.3	446.7	893.4	1786.8	3573.5	7147.0
Zn (nM) in CER Euglena	0	0.68	1.38	2.75	5.51	11.01	22.03	44.06
Zn (nM) in CER Euglena + Zn	0	111.6	223.3	446.7	893.4	1786.8	3573.5	7147.0

## Data Availability

The datasets analyzed during the current study are available from the corresponding author on reasonable request.
